# Supplementary material for: Are carbon emissions trading and green financial instruments synergistic? -Comprehensive quantitative research based on content analysis
Source: PLoS One. 2024 Mar 7;19(3):e0298601. doi: 10.1371/journal.pone.0298601 (PMC10919601; doi:10.1371/journal.pone.0298601)
Supplement: S1 Data — (ZIP) [file pone.0298601.s001.zip › Data/地级市Z_F工作报告.zip!/║╙─╧/╚2├┼╧┐╩╨/└┤╫╘╣1⁄2╓┌║┼í╛┬φ┐╦╩2╛▌═°í┐_2012.docx]

各位代表：

　　现在，我代表市人民政府向大会报告工作，请予审议，并请各位政协委员提出意见。

　　一、2011年及本届政府工作回顾

　　2011年，是全市科学发展、加速转型的一年。我们抢抓中原经济区建设机遇，紧紧围绕“三大战略定位”和“十六字”目标，深入推进“四大一高”战略，顺利完成了市五届人大六次会议确定的各项任务，实现了“十二五”开门红。2011年，全市生产总值完成1062.9亿元，比上年增长12.8%。全社会固定资产投资完成760.9亿元，增长24.8%。地方财政一般预算收入完成57.6亿元，增长15.8%。城镇居民人均可支配收入17062元，增长13.5%；农民人均纯收入6929元，增长19.7%。一年来，主要做了七个方面的工作：

　　（一）着力优化结构，发展质量持续提升。坚持新型工业化为主导，全市规模以上工业完成增加值640.1亿元，比上年增长16.1%；高新技术产业增加值增长15.3%。巩固农业基础地位，粮食产量保持稳定，果品总产量18亿公斤，居全省之首，雏鹰农牧等项目开工，“函谷红” 茶和“三门峡”卷烟上市，特色农业占农业总产值的82%。繁荣现代服务业，大中海商业广场等大商贸项目进展顺利，社会消费品零售总额完成239.3亿元，增长18.1%；全年接待游客1474万人次，实现旅游收入117亿元，分别增长20.5%和30.6%。县域经济发展迅速，各县（市）在全省综合排名实现晋位。

　　（二）着力项目带动，发展后劲显着增强。继续深化项目建设年活动，开祥化工1,4—丁二醇一期等90个项目建成投产，重点项目投资额、建成数双双创历史新高；省重点项目联审联批事项提前5个月完成全年任务，居全省第一，被评为2011年度全省重点项目建设管理先进单位、企业服务先进单位。加快产业集聚区发展，全市7个产业集聚区实施项目218个，完成投资279.9亿元，规模以上工业实现主营业务收入1026.6亿元。三门峡产业集聚区被评为省“十快” 产业集聚区、 “省级高新技术开发区”，义马煤化工产业集聚区被评为省先进产业集聚区。

　　（三）着力城乡建设，发展基础日益稳固。蒙西铁路获国家发改委批复，三灵快速通道、三淅高速等大交通项目进展顺利。城市空间发展战略研究通过专家评审，新一版城市总体规划正在编制。中心商务区、黄河公园、旧城区和城中村改造等重点工程稳步推进。北环路建设启动，城市路网更加完善。集中供热二期续建工程完工，新建热交换站11座，新增供热面积80万平方米，改造旧管网3.5万米。城市精细化管理扎实推进，林业生态建设走在全省前列，国家森林城市创建通过验收。全市112个新型农村社区新建住宅1.03万套，累计建成1.87万套。

　　（四）着力扩大开放，发展活力更加强劲。积极推动大通关建设，三门峡海关大厦主体封顶，三门峡出入境检验检疫局正式运行，国家铝及铝制品质量监督检验中心正在安装检测设备。全年出口总额1.6亿美元，比上年增长55.9%。深入开展大招商，成功举办第17届旅游节暨投洽会，参加中博会等重大经贸活动，共签约项目110个，总投资突破1000亿元，全市实际利用外资6.3亿美元，总量居全省第三。深化央企合作，与中国黄金集团等10家央企签订战略合作协议，签约金额207.6亿元，居全省第四。

　　（五）着力破解瓶颈，发展要素保障有力。节约集约利用资源，整理、整治土地3042.3公顷；煤矿兼并重组基本完成，省定矿产资源整合通过验收；小秦岭深部探矿和渑池煤下铝整装勘察项目列入国家找矿计划，灵宝—卢氏矿集区列入国家矿产资源综合利用示范基地。拓宽融资渠道，在省银企合作会议上签约项目67个，总投资177亿元，规模居全省第二；组建两大投资集团，市财经投资公司发行债券15亿元。硬起手腕整治环境，关停重金属污染企业55家，启动农村环境连片综合整治项目11个，铅污染治理和铬渣处置工作有序推进。

　　（六）着力改善民生，发展成果惠及群众。扎实办好民生实事，省、市“十大民生工程”全面完成。城镇新增就业5.96万人，城镇登记失业率3.24%，低于省控标准。社会保障体系逐步完善，城乡低保实现了动态管理下的应保尽保。开工保障性住房8367套，两个廉租房小区被评为“全省十佳住房保障工程”。加快校安工程建设，中小学D级危房全面消除。基本药物制度覆盖村级卫生室。新农合参合率98.7%，财政补助标准提高到200元，报销封顶线提高到10万元，减轻了群众看病负担。文化体育事业快速发展，陕县西张村镇、灵宝阳平镇被命名为“中国民间艺术之乡”，122个村建成“农民体育健身工程”。社会管理创新成效显着，被授予“中原平安杯”，连续两届荣获“全国社会治安综合治理优秀市”称号。

　　（七）着力转变职能，政府自身建设成效显着。深入开展“两转两提”，依法行政工作扎实推进。主动接受市人大及其常委会监督，支持市政协履行职能，认真办理人大代表建议75件、政协委员提案193件。第七届村委会换届圆满完成。深化行政审批制度改革，取消、下放行政审批项目12项。应急保障体系逐步完善，妥善处置“陇海铁路山体滑坡”等突发事件。全面实施“六五”普法和依法治理工作规划。廉政建设和反腐败斗争深入推进。思想道德和精神文明建设不断加强。

　　国防教育和国防后备力量建设取得新成效，实现了国家双拥模范城“四连冠”，驻三门峡解放军、武警部队和广大预备役人员在支持地方经济建设、维护社会稳定等方面作出了重要贡献。统计、审计、民族、宗教、外侨、对台、档案、史志、广电、体育、人防、气象、无线电、防震减灾、黄河河务、妇女儿童、残疾人、老龄等工作取得新成绩。对口援疆工作进展顺利。

　　2011年各项任务的完成，标志着本届政府胜利实现了市五届人大一次会议确定的任期目标。本届政府任期以来，面对金融危机的严峻考验，面对洪涝等自然灾害的袭击，面对保增长与调结构的双重压力，面对形势复杂多变、资源环境约束、结构性矛盾加剧等各种挑战，全市上下凝心聚力，克难攻坚，办成了一批具有深远意义的大事，办好了一批深受群众欢迎的实事，办妥了一批前所未有的难事，在逆境中实现了加速转型，在困难中保持了较快发展，在拼搏中铸就了新的辉煌！

　　五年来，我们化危为机、逆势而上，综合实力实现跨越。在国际金融危机的冲击面前，见事早、行动快，果断采取“一体两翼” 经济刺激计划和“过冬五策”，鼓励企业“抱团取暖”，在逆境中保持了昂扬的发展态势。2011年，全市生产总值是2006年的2.6倍，年均增长14.5%；地方财政一般预算收入是2006年的2.7倍，年均增长 21.6%；全社会固定资产投资是2006年的3.3倍，年均增长27.1%；社会消费品零售总额是2006年的2.4倍，年均增长19.2%。

　　五年来，我们调结构、促转型，科学发展路径明确。抢抓“后危机时代”机遇，针对“一产不优、二产不强、三产不大”等问题，科学确定“三大战略定位”和 “十六字” 目标，提出“四大一高”战略，闯出了一条转型发展的新路子。五大支柱产业增加值占规模以上工业增加值的77.5%；五年累计节能260万吨标准煤，万元GDP能耗比2006年下降23.9%。

　　五年来，我们锐意改革、扩大开放，创新活力充分涌流。始终把改革开放作为保增长、促转型的关键性举措，国有企业、政府机构、医药卫生、财税金融等改革深入推进。对外开放不断扩大，大通关体系建设初见成效，旅游节暨投洽会成功升格为国家级节会活动，五年累计实际利用外资17亿美元，2011年实际利用外资是2006年的4.8倍。自主创新体系日益完善，全市建成省级院士工作站7家、省级研究中心31家，科技进步对经济发展的贡献率提高到53.9%。

　　五年来，我们加快建设、提升功能，城乡面貌焕然一新。深入实施中心城市带动战略，拉大城市框架，提升城市品位，完善城镇体系，加快新农村建设。中心城市建成区面积拓展5平方公里，建成了高铁南站、国际文博城、天鹅湖湿地公园等一批重点工程，城市在“长高”，家园在“变靓”，相继荣获“国家园林城市”、“中国大天鹅之乡”等称号。城镇建设步伐加快，城镇化率由40.8%提高到46%。农业和农村工作扎实推进，农业信息化全国领先，农村经济持续发展。

　　五年来，我们给力民生、情系百姓，群众得到更多实惠。始终把保障和改善民生作为第一追求，坚持每年实施“十大民生工程”，以改善民生凝聚民心、汇聚民力。2011年，城镇居民人均可支配收入是2006年的1.9倍，农民人均纯收入翻了一番。五年累计向农民发放各类补贴22.2亿元，城市引水、集中供热、市外语高中等一大批民生工程惠及百姓。文化事业蓬勃发展，公共文化基础设施覆盖率达80%以上。五年累计新增城镇就业30万人，城、乡低保人均月补差分别提高113元、53元，解决了近60万农村人口的安全饮水问题。

　　各位代表！回顾这浓墨重彩的五年，我们为三门峡发生的巨大变化而骄傲；回首这砥砺奋进的岁月，我们为全市人民创造的辉煌业绩而自豪！实践证明，勤劳、淳朴、厚道的三门峡人民能干事、会干事，更能干成事、干成大事！

　　各位代表！回首这战危机、谋发展的艰辛历程：我们难以忘记，在应对危机的关键时刻，省委、省政府和市委悉心关注，给予我们的坚强领导；我们难以忘记，在保增长、促转型的攻坚阶段，各位离退休老干部、老同志出谋划策，给予我们的关心和帮助；我们难以忘记，在重点项目和基础设施建设的拆迁过程中，广大群众舍小家为大家，给予我们的理解和支持；我们难以忘记，在政府工作的高效推进中，人大代表、政协委员积极监督、参政议政，给予我们的宝贵意见和建议；我们难以忘记，在艰巨繁重的任务面前，全市人民同舟共济、众志成城，付出了超乎寻常的艰苦努力。在此，我代表市人民政府，向工作在全市各条战线上的工人、农民、知识分子、人民解放军指战员、民兵预备役人员、武警官兵、公安民警以及社会各界人士表示衷心的感谢！

　　各位代表！艰难困苦，玉汝于成。五年来的拼搏与奋斗，特别是战危机、保增长、促转型的生动实践，让我们越来越深刻地体会到：加快三门峡发展，必须突出大通关。以大通关建设为平台，铺就外贸进出口“高速路”，开启思想解放的大门，增创对外开放新优势。加快三门峡发展，必须突出大交通。坚持交通先行，密切与各大经济板块的联系，连接金三角，融入大中原，增创区位环境新优势。加快三门峡发展，必须突出大商贸。以大商贸拉动内需，扩大消费，繁荣市场，全面提高三门峡的辐射力，增创区域竞争新优势。加快三门峡发展，必须突出大旅游。以文化旅游为载体，培育经济发展新支柱，塑造城市品牌，扩大对外影响，增创持续发展新优势。加快三门峡发展，必须突出高新技术产业。倾力推进传统产业高端化、新兴产业规模化，以科技推动转型，以创新引领未来，增创转型发展新优势。加快三门峡发展，必须突出“四个基层”。坚持着眼基层，依靠基层，着力基层，服务基层，密切联系群众，弘扬务实重干，为推进“四大一高”战略提供坚强保障。这些经验和体会，是启迪，是探索，更是宝贵财富，在今后的工作中，我们必须一以贯之、完善提升、发扬光大！

　　在看到成绩的同时，我们也清醒地认识到，当前政府工作仍面临不少困难和压力：经济转型的任务还很繁重，资金、土地、环境等要素制约需尽快破解，新型城镇化引领作用发挥不够，持续改善民生的任务依然艰巨，政府自身建设还存在不少薄弱环节等。对此，我们一定高度重视，保持清醒和忧患，采取有力措施加以解决。

　　二、今后五年政府工作的总体要求和奋斗目标

　　未来五年，是我市打造中原经济区重要支撑、全面建设小康社会的关键期，也是加速经济结构调整、加快发展方式转变的攻坚期。虽然国际经济环境还很复杂，但我国的发展仍处在重要战略机遇期。中央确定稳中求进的总基调，实施积极的财政政策和稳健的货币政策，着力实体经济发展；省委、省政府对我市打开“西大门”、建好“西大门”寄予厚望，中原经济区建设如火如荼，黄河金三角区域协作深入推进；市六次党代会描绘了宏伟蓝图，“十二五”发展开局良好，全市上下谋发展、促转型的热情高涨。只要我们抢抓历史机遇，坚持第一要务，加速转型发展，就一定能够在新一轮的发展中点燃激情、成就梦想、创造辉煌！

　　未来五年政府工作的总体要求是：抢抓加快建设中原经济区的历史机遇，紧紧围绕“三大战略定位”，强力推进“四大一高”战略，以科学发展为主题，以经济转型为主线，以“三化”协调发展为统领，以重点项目、产业集聚区、农业产业集群、城乡建设、招商引资为抓手，着力扩需求，着力创优势，着力破瓶颈，着力惠民生，进一步促进全市经济在总量上扩张、结构上优化、质量上提升，加快建设创新开放、富裕文明、平安和谐、生态宜居三门峡。

　　今后五年的主要奋斗目标是：

　　——加速转型转变，建设创新开放三门峡。“四大一高”优势凸显，转型升级实现突破，“三化”协调发展，科技进步对经济增长的贡献率达56%以上，改革开放深入推进，努力建成二类口岸作业区，年度进出口总额超过3亿美元。

　　——实现经济翻番，建设富裕文明三门峡。全市生产总值、财政收入、工业增加值、固定资产投资等主要经济指标增速高于全省平均水平，领先黄河金三角地区；经济总量和公共财政预算收入力争五年翻一番。坚持富民与强市并重，确保城乡居民收入同步增长。

　　——创新社会管理，建设平安和谐三门峡。五年城镇新增就业20万人，转移农村剩余劳动力35万人；实现社会保险制度全覆盖，社会保障体系更加完善；城乡居民生活质量明显改善，各项社会事业均衡发展，社会管理体系更加健全，平安建设继续走在全省前列。

　　——统筹城乡发展，建设生态宜居三门峡。中心城市组团发展迅速，城市新区建设全面启动，中心商务区、特色商业区形象显现，新型农村社区惠及更多群众；城镇化率达到53%左右。节能减排工作深入推进，万元GDP能耗进一步下降，城市环境空气质量优良天数达到320天以上；全市森林覆盖率达到50.5%，让城乡处处充满绿色和生机。

　　三、2012年政府工作的主要目标和任务

　　按照市委六届三次全会暨市委经济工作会议“求转、求进、求实”的总要求，今年经济社会发展的主要预期目标是：地区生产总值增长12%以上；公共财政预算收入增长13%以上；规模以上工业增加值增长16%以上；全社会固定资产投资增长22%以上；社会消费品零售总额增长16%以上；实际利用外资增长20%以上；外贸出口总额增长20%以上；城镇居民人均可支配收入和农民人均纯收入增长9%以上；城镇登记失业率控制在4.5%以内；人口自然增长率控制在6.5‰以内；万元GDP能耗下降3.7%左右。

　　围绕总体要求和今年主要目标，重点抓好六个方面工作：

　　（一）强力推进“四大一高”战略，加快经济发展方式转变。把“四大一高”战略作为加快经济发展方式转变的主要抓手、实现“三化”协调的重要载体、提升综合实力的强大引擎，坚定信心，强力推进。

　　完善大通关，打造内陆开放高地。加快通关平台建设。强力推进三门峡海关物流中心建设，抓紧规划建设海关监管查验场所等配套设施。积极做好综合保税区的申报工作，国家铝及铝制品质检中心年底前投入运行。稳步扩大果汁等传统产品出口，提高机电等高新技术产品的出口比重。鼓励企业培育国家级出口品牌，支持各县（市、区）争创省级出口基地。拓宽对外开放领域。加快服务业对外开放，吸引境内外投资者进入物流、金融、文化、旅游等领域。降低准入门槛，鼓励支持民间资本投资社会事业。强力实施招商引资。突出资源和产业优势，认真组织项目推介和重大招商活动。研究产业转移的趋势和特点，派出招商小分队，紧盯京津唐、长三角、珠三角、西安等重点区域驻地招商。依托产业上下游，开展产业链招商；围绕企业供销链，实行以商招商，力争全年利用外资7.6亿美元，利用省外资金200亿元。开展“招商引资项目落地年”活动，提高招商引资实效。继续深化央企合作。认真研究央企发展战略，建立与央企合作的长效机制，推动大唐100万千瓦机组等项目早日投产。加快推进中金集团黄金产业园、国电灵宝2×30万千瓦机组等项目前期工作，确保中金集团黄金产业园年内开工。主动融入区域经济板块。继续做好“黄河金三角地区承接产业转移示范区”申报工作。完善“郑洛三”工业走廊规划，积极争取省政府出台支持“郑洛三”工业走廊的措施。谋划与洛阳等周边城市的合作对接，构建豫西经济板块。

　　提速大交通，打造地区交通枢纽。突出“三纵四横”建设。三淅高速卢氏县城北段、郑卢高速洛宁至卢氏段年底前建成通车；连霍高速改扩建、三淅高速卢氏县城南段全面掀起施工高潮；做好蒙西铁路三门峡段、运宝高速、渑垣高速的前期工作。优化市域路网结构。加快中心城市与各县城之间的通道建设，打通“断头路”，拓宽“瓶颈路”。三灵快速通道年底前全线通车，完成农村公路改建200公里，公共交通逐步向农村延伸，让老百姓出行更方便。

　　繁荣大商贸，打造黄河金三角商贸、物流、金融中心。繁荣商贸服务业。加快推进义乌国际商贸城等项目，确保大中海商业文化广场年内开业；积极引进国内外知名商贸企业，努力建设黄河金三角区域性商贸中心。加快发展现代物流业。依托高铁南站和三门峡海关，培育综合物流。依托三门峡西站，规划建设3000万吨煤炭物流中心。依托连霍高速公路改扩建，科学规划物流节点。抓好金秋果业等果品物流项目，建设食品冷链物流园区。支持七海物流等企业做大做强，积极引进国内外大型物流集团。促进金融业与物流业合作，发展质押监管等金融物流，努力建设黄河金三角区域性物流中心。大力发展金融服务业。积极引进股份制银行，加快发展证券、保险等金融业务，形成功能互补、充满活力的金融服务体系，努力建设黄河金三角区域性金融中心。

　　发展大旅游，打造旅游经济强市。科学修编三门峡旅游业发展总体规划，强化沿黄九省和黄河金三角地区旅游合作，叫响“黄河之旅”旅游品牌。深度挖掘仰韶文化、老子文化、虢国文化，高层次开发温泉资源。抓好天鹅湖湿地公园、函谷关历史文化旅游区5A级景区创建，加快三门峡游客服务中心建设，努力成为“王朝街道”之旅的重要旅游目的地。以郑州、西安、太原“大三角”区域为主要客源地，科学定位旅游产品，加强旅游宣传，扩大对外影响。精心筹办第18届旅游节暨投洽会，树立三门峡旅游形象。

　　壮大高新技术产业，加快技术创新步伐。着力推进高新技术项目。速达电动汽车等13个高新技术产业项目，年内完成投资34亿元以上。抓好亚熔盐法氧化铝清洁生产、8—12微米高档电解铜箔等国家级科技项目，加快推进高效数控机床等5个部级科技项目。提高企业自主创新能力。利用义煤集团、华鑫铜箔等优势企业的技术创新平台，加强与高等院校、科研院所的合作，新建省级院士工作站2个。鼓励企业加大科研投入，支持国家铝及铝制品质检中心、河南煤化工质检中心提升服务水平。实施科技人才引进计划，完成星火培训5.6万人。

　　坚持项目带动，为推进“四大一高”战略注入强大动力。确定市级重点项目221个，总投资2010亿元，年计划完成投资400亿元以上。加快重点项目进度，确保义煤综能1000万标方煤制气一期等80个项目投产；河南煤化汽车零部件产业园等108个新开工项目，年内完成投资100亿元以上。加强项目储备，亿元以上项目储备保持在200个以上。

　　（二）突出新型城镇化引领，建设宜业宜居家园。加快城市组团发展。科学修编城市总体规划，打造以湖滨城区、开发区和中心商务区为基础的老城区，以陕县县城、产业集聚区为基础的新区“两大”组团；以义渑城区、灵宝市区和卢氏县城为主体的“三小”组团，形成城市组团联动发展格局。完善中心城区功能。全面建成国际文博城，加快传媒大厦、文化公园等城市重点工程进度。年内完成黄河公园道路、广场、绿化和服务设施主体工程，推进沿黄半岛景观带建设。加快旧城区和城中村改造。北环路年底建成通车。强化城市管理，将精细化管理落实到城市的每一个角落。理顺新区管理体制，拓展城市发展空间。加快建设中心商务区。积极做好中心商务区发展规划申报，争取在全省率先获批；完善中心商务区基础设施专项规划、节点景观修建性规划。全面铺开基础设施建设，供水工程上半年通水，年内建成8条城市道路、启动4座桥梁和苍龙涧河公园工程。做好项目招商，年内落地项目15个以上。按照“一年打基础、两年大变化、三年成规模”的总体要求，争取用3年时间，把中心商务区建成集商务办公、金融会展、商贸流通、旅游集散、文化娱乐、休闲购物为一体的高端商务区，成为辐射黄河金三角地区的高端商务中心。科学规划特色商业区。抢抓政策机遇，科学规划建设特色商业区，每个特色商业区年内新开工服务业项目3个以上，打造现代服务业集聚发展平台。持续加快新型农村社区建设。把新型农村社区建设作为“三化”协调的突破口，抓紧完成全市新型农村社区布局规划；引导农民在规划完善、公共设施健全的地方建房；新开工建设住宅1万套，实现农民就地就业、合理集聚。

　　（三）强化新型工业化主导，提升经济转型的支撑能力。以支柱产业、关键项目为支撑，积极推进产业、产品结构和企业组织结构调整，建设“郑洛三”工业走廊，努力成为区域发展的重要增长极。加快传统产业高端化。用先进技术改造提升主导产业，实现主导产业的延链、补链、强链。铝工业，做好铝的精深加工，围绕铝箔、型材、高档铝合金炊具、大截面高性能铝型材等产品，积极向下游延伸，抓好华晟铝业10万吨高档炊具合金铝板等项目，加快同人铝业60万吨铝铸件项目前期工作。统筹发展非铝产业，推进双旭科技5万吨拟薄水铝石等项目。煤化工产业，向产业链高端发展，抓好义煤综能1000万标方煤制气、开祥化工10万吨PTMEG（聚四亚甲基醚二醇）等项目，积极发展二甲醚等清洁燃料。黄金及有色金属工业，依托中金集团黄金产业园、金源矿业1万吨压延铜箔等项目，抓好高档黄金饰品的设计加工，做好黄金伴生金属的综合回收、精深加工。能源产业，协调义煤集团，加快整合煤矿的技术改造和复产，确保煤炭年产量2000万吨以上，渑池2×30万千瓦机组项目年内通过核准，加快推进燕山风电场等项目建设。食品工业，重点向终端消费品方向发展，抓好缘份果业15万吨矿泉水和果蔬饮品、景源果业10万吨果沙饮品、尚正食品50万吨冷链物流等项目，培育知名品牌。加快发展果蔬饮料、果酒、果醋、果胶等果品精深加工。装备制造及汽车零部件产业，重点做好专用设备和汽车零部件提质增量，积极发展成套设备。抓好骏通5万辆专用车辆销售，加快推进1万辆汽车起重机项目。推进博市电器200万台冰箱、200万台洗衣机等项目，做好新华水工机械公司迁建项目前期工作。加快新兴产业规模化。新能源汽车，抓好速达纯电动汽车及配套项目。新材料产业，抓好义腾新能源锂电池隔膜二期等项目，积极谋划金渠集团超硬材料公司扩建项目。生物医药产业，抓好孟成制药2000吨L-色氨酸、北京嘉林药业赛诺维制药等项目。光伏及新能源产业，抓好华光新能源300万片蓝宝石抛光片等项目，发展光伏及光伏玻璃产业链。加快产业集聚区建设。进一步完善基础设施，力争年内完成投资72亿元以上，各集聚区污水处理厂年底前基本建成。围绕主导产业，推动产业纵向连接、侧向配套，实施亿元以上的转型升级项目55个。深入推进产城融合，力争集聚区年内改造村庄16个，实现公交、邮政等市政服务全覆盖。加快实施“产业集聚区和城镇化建设百亿筹资计划”，做大做强融资平台；加强土地整治、节约集约、监督管理三项机制的落实，提升土地保障能力。年底各产业集聚区力争完成固定资产投资380亿元以上，实现主营业务收入1300亿元，分别占全市的40%以上。着力培育优势骨干企业。支持优势企业壮大规模，确保全市主营业务收入超百亿元的工业企业达到3家以上，超50亿元的7家以上，超10亿元的35家以上。加快推进企业上市，争取更多企业进入省上市“后备队”。努力打造知名品牌。引导企业强化质量意识，加大品牌创建力度，培育和扶持发展一批名牌产品。抓好工业运行调节。强化经济运行监测，密切关注铝工业等重点行业、企业的运行态势。抓好煤电油运要素保障，积极做好电煤保供，完善电力需求侧管理。做好已投产工业项目的调试，促使尽快达产达效，形成新的增长点。全力做好企业服务，帮助企业减轻负担，破解难题。

　　（四）夯实新型农业现代化基础，大力促进农民增收。加大强农惠农富农力度，在稳定粮食生产的前提下，大力发展现代特色农业，促进农业增效、农民增收。打造现代特色农业集群。发展果品出口基地20万亩，果品总产超过18亿公斤。建设生态养殖、屠宰加工基地，肉类产量8.2万吨。完成造林35.3万亩，种植烟叶27万亩，新发展食用菌2000万袋。高度重视“菜篮子”工程，蔬菜总产量8.8亿公斤。推进茶叶基地建设，开发“函谷红”中药保健茶。整合现有资源、基地和龙头企业，打造优质果品、高档牛肉、食用菌、烟叶等特色农业集群。提高农业产业化水平。加大扶植力度，力争各类规模以上农业龙头企业达到170家以上。用资本理念指导涉农企业上市，培育仰韶生化等涉农企业上市“后备队”。提高农业生产组织化程度，农民专业合作社发展到390家以上。推进农业科技创新。加强特色农业技术研究，力争农业信息化等技术达到国内先进水平。健全农业标准化生产技术体系，新认证无公害农产品、绿色食品、有机食品、地理标志保护农产品20个。加快农业信息化建设，积极推广农业物联网技术。改善农村生产生活条件。加快农田水利基本建设，完成病险水库除险加固年度任务，陕县苍龙涧河贺家庄段等7个河道治理项目年底前竣工，抓好卢氏县焦子河等小流域坝系工程，做好大石涧水库等项目前期工作。完善农村基础设施，加快农村饮水安全工程建设，新建农村户用沼气4500户，再帮助1.8万贫困人口脱贫致富。

　　（五）大胆创新体制机制，绽放发展的激情与活力。加快重点领域和关键环节的改革，让科学发展的激情与活力全面迸发。深化文化体制改革，促进文化大繁荣。推进文化企事业单位、文化市场综合执法改革。加快老子文化产业园、仰韶文化产业园等项目建设，着力开发虢国澄泥砚等文化旅游商品。推进其他领域改革。全面开展农村集体土地确权登记，全市农村土地流转面积达到40万亩。深化财税体制改革，稳妥推进公务卡和预决算公开制度。深化行政审批制度改革，进一步取消、调整、规范行政审批项目，继续清理行政事业性和经营服务性收费，优化经济发展环境。推进事业单位、医药卫生体制改革。加快非公有制经济发展。落实各项政策措施，完善小微企业服务体系，促进非公有制经济健康发展。创新投融资体制机制。落实政银战略合作协议，争取信贷资金投放增幅高于全省平均水平。全力争取政策性资金，做好资本市场融资，支持企业上市。鼓励市商业银行在市外设立分支机构，支持农信社组建农村商业银行，规范整顿中小企业融资担保机构。创新土地管理机制。严格执行供地政策和用地标准，争取更多项目列入省重点项目管理。强力推进土地综合整治，实现占补平衡有余。完善节能减排机制。抓好重点领域节能减排，做好城市污水处理厂规划，现有生活污水处理厂年底前全部改造完毕。深入推进大气污染综合整治，抓好重点河流污染监测和治理。巩固铅污染治理成果，全面完成义马铬渣处置任务。抓好农村环境连片综合整治。创新资源利用和保护机制。加强矿产资源储量的动态管理，节约利用资源。加大煤下铝、小秦岭金矿等整装勘察和深部探矿力度，为发展提供后备资源。

　　（六）扎实办好民生实事，让人民群众享受发展成果。认真落实好省、市“十大民生工程”。千方百计扩大就业。落实结构性减税和信贷优惠政策，全力支持小微企业发展，创造更多的就业岗位；实施“崤函创业扶持行动”，全年发放小额担保贷款5亿元以上。加强就业培训，全年新增就业4.2万人。密切关注群众就医。实施国家基本药物制度，完善全民医保体系，城镇居民医保和新农合补助标准提高到每人每年240元。开展“健康快车”项目，免费实施白内障复明手术1500例。努力促进教育公平。加大教育投入，科学编制基础教育规划，实施“学前教育三年行动计划”，重点推进市实验中学等11所校舍安全工程项目建设。加强校车安全管理，让孩子们平安上下学。整合职业教育资源，加快职教园区建设，扎实抓好本科院校创建。加快保障性安居工程建设。新开工保障性住房10923套，年底竣工7200套；制定和完善保障性住房建设、分配、管理、退出机制，努力使低收入群体住有其居。切实加强社会保障。加快社会保障体系建设，城乡居民社会养老保险制度年底实现全覆盖，为实现老有所养奠定基础；加强社会救助标准与物价上涨联动机制建设，保证救助群众生活水平不降低。大力发展社会福利、养老、慈善、妇女儿童和残疾人事业。继续做好人口和计划生育工作，稳定低生育水平。扩大基本公共服务。优先发展公共交通，优化公交线路，更新66台公交车，继续推行70岁以上老人免费乘车，对60—69岁老人乘车实行半价优惠。加快城区集中供热的新热源建设和管网改造，进一步提高供热质量和覆盖面，让群众温暖过冬。繁荣文化体育事业。继续实施基础文化设施覆盖工程，为老百姓送上更多的文化“大餐”；做好崤函古道等申遗工作，加强文物、非物质文化遗产保护。广泛开展全民健身运动，不断提升竞技体育水平。抓好安全生产和食品安全。从严落实安全生产责任，防范和遏制重特大事故。整合管理资源，坚持最严的准入、最严的执法、最严的处罚、最严的问责，加强食品安全监管，让广大群众吃的放心。深入推进社会管理创新，巩固全国“试点市”建设成果。加强社会治安综合治理，积极排查和化解社会矛盾。

　　支持驻峡解放军、武警部队建设，抓好国防教育、国防动员、人民防空和民兵预备役工作，深入开展双拥共建活动，巩固军政、军民团结。切实做好统计、史志、外侨、应急管理、黄河河务、机关事务、防震减灾、广电、民族、宗教、气象等工作。继续做好援疆工作。

　　四、加强政府自身建设

　　复杂多变的经济形势，艰巨繁重的发展任务，对政府工作提出了新的更高要求。我们将继续用领导方式转变加快发展方式转变，认真落实“四个重在”实践要领，坚持“三具两基一抓手”工作方法，深入推进“两转两提”，树立推动工作、为民负责、清正廉洁的政府形象。

　　增强法治观念，提高依法行政能力。加强法治政府建设，认真执行人大及其常委会的决议、决定，依法接受人大及其常委会的法律监督、工作监督和人民政协的民主监督，提高建议提案办理质量。充分发挥监察、审计等专门监督的作用，健全行政监督体系。完善电子政务系统，健全行政服务体系，真正做到高效、便民。

　　增强效能观念，提高工作执行能力。弘扬崇尚实干、埋头苦干的精神，快节奏、高效率、大力度抓好工作落实。坚持“学明白、想明白、说明白、做明白”，说到做到，说好做好。建立责任机制，加强行政效能监督，治庸、治懒、治散、治软。

　　增强学习观念，提高科学运作能力。倡导全员学习之风，打造学习型政府。完善重大行政决策程序规定，向专家求教，拜群众为师，使各项措施更加符合实际、经得起实践检验。善于整合资源，凝聚力量，调动一切积极因素，集中推进重点工作、重点项目。主动听取各民主党派、工商联、各人民团体及社会各界人士的意见建议，努力形成共同推动经济社会发展的强大合力。

　　增强党性观念，提高廉洁从政能力。自觉加强党性修养，认真贯彻廉政准则，落实党风廉政建设责任制，强化权力运行的制约和监督，让权力在阳光下运行。推进反腐倡廉制度创新，强化岗位廉政风险防控。大力弘扬艰苦奋斗精神，坚持勤俭节约，反对铺张浪费，严格控制行政经费等一般性支出，把有限的资金用在为老百姓办实事的“刀刃”上。

　　各位代表！我们正站在继往开来的历史起点上，新的征程已经开启，新的目标催人奋进。让我们在省委、省政府和市委的坚强领导下，在市人大、市政协的监督和支持下，围绕“三大战略定位”，强力推进“四大一高”战略，用团结凝聚力量，用务实加快发展，努力在中原经济区建设中奋发有为，以优异成绩迎接党的十八大胜利召开！
